# Management dilemma of a recurrent huge fibrosarcoma in a 25-year-old African: a case report

**DOI:** 10.4076/1752-1947-3-7267

**Published:** 2009-06-11

**Authors:** Ismaila A Adigun, Kolawole O Ogundipe, Jibril O Bello

**Affiliations:** 1 Division of Plastic and Reconstructive Surgery, Department of Surgery, University of Ilorin Teaching Hospital, Ilorin, Nigeria

## Abstract

**Introduction:**

Soft tissue sarcomas are rare tumours that are infrequently seen in most surgical practices. They can occur in any anatomic region of the body. The size of a soft tissue sarcoma is an important prognostic variable and so affects the quality of tumour resection.

**Case presentation:**

A 25-year-old Nigerian African presented with recurrent huge fibrosarcoma measuring about 55 × 40 × 10 cm at his posterior trunk. The patient's clinical condition was poor; the tumour seemed unresectable and the patient looked inoperable. He had an extensive excision of the tumour but could not afford adjuvant therapies. He was discharged home against medical advice but may succumb to metastases.

**Conclusion:**

Sarcomas in black people can present as extremely large masses; the dilemma in management is not only limited to the delay in presentation but also the poor socio-economic status of the patients and the frequent non-availability of supporting services. Treatment grants or subsidies from government may go a long way to ensuring that patients receive appropriate care.

## Introduction

Soft tissue sarcomas (STS) are rare tumours that are infrequently seen in most surgical practices. They arise from mesodermally derived extra-skeletal tissues which constitute more than 50% of the body weight. The tumour accounts for about 0.7% of all adult malignancies but up to 15% of childhood malignancies [1]. Soft tissue sarcomas can occur in any anatomic region of the body because of the ubiquitous nature of connective tissue, but most sarcomas develop in the extremities. The size of a STS is an important prognostic variable and affects the quality of tumour resection. Achievement of an R0 resection (tumours completely surrounded by uninvolved tissue) initially could be a very important part of multidisciplinary therapy. We report a 25-year-old Nigerian who presented with a huge recurrent tumour having had three previous excisions of a soft tissue tumour of the trunk.

## Case presentation

The patient was a 25-year-old farmer who presented to our unit with a painless mass on his back of 13 years duration. The mass had been increasing in size. There was associated weight loss, cough and features suggestive of chronic anaemia. He had had three previous excisions of the mass at a peripheral hospital and on each occasion, the excised specimen had been discarded.

Examination revealed a young man, pale and with a pulse rate of 100 bpm. He had a fungating mass on his posterior trunk extending from inferior angles of the scapula to about 10 cm above the iliac crest, the mass measuring about 24 × 16 × 5 cm. His chest was clinically clear and with no palpable organomegaly. An assessment of soft tissue sarcoma was made. The patient was resuscitated and transfused with 2 pints of blood. A wide excision of the mass was performed. Postoperative radiotherapy was planned before wound cover with split thickness skin graft (STSG). However, the patient discharged himself against medical advice. Histology of the excised specimen confirmed fibrosarcoma.

He represented about 7 months later with a similar mass on his back. The mass covered almost the entire posterior trunk, bled with the slightest contact, and restricted his mobility. He had features suggestive of anaemic heart failure; he was pale, had a pulse rate of 130 bpm, bounding and had an audible third heart sound. He was also wasted and in respiratory distress.

**Figure 1:**
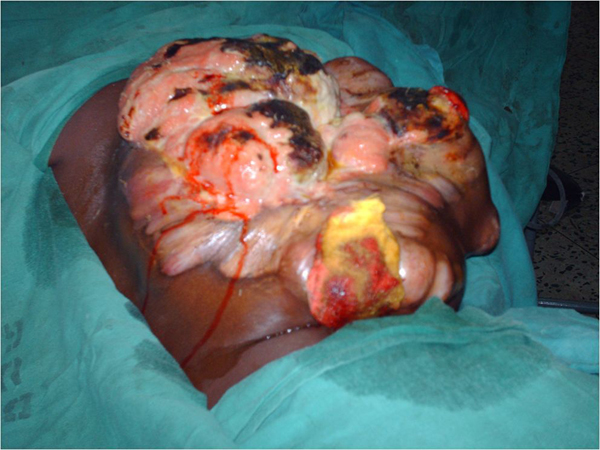
**Pre-operative status of the patient**. The figure shows the patient on the operating table, already draped, and showing the massive tumour at his posterior trunk.

**Figure 2:**
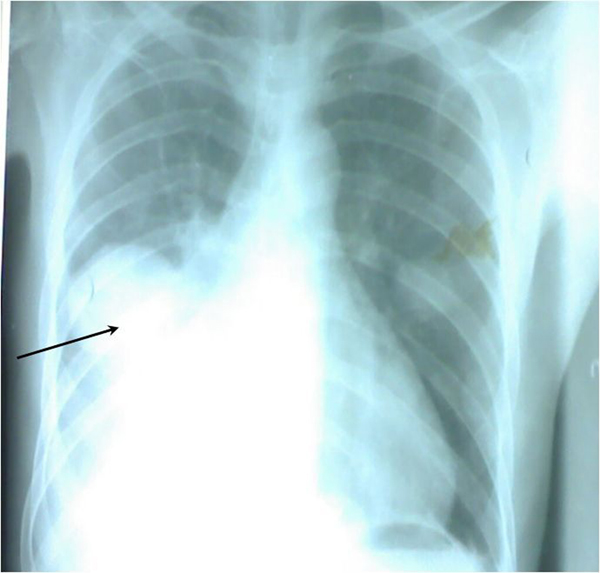
**Pre-operative chest X-ray of the patient**. A postero-anterior X-ray film of the patient's chest taken just before surgery showing cannonball metastasis and pleural effusion in the right lung. The arrow indicates the cannonball metastasis.

**Figure 3:**
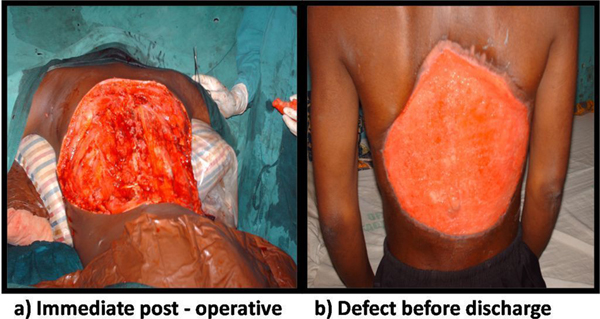
**Postoperative status of the patient**. **(a)** The defect at his posterior trunk immediately postoperative. **(b)** The wound had contracted significantly at the time of discharge against medical advice 4 weeks postoperatively. Some nodules of progressive tumour can also be seen along the spine.

A huge fungating mass was present measuring 55 × 40 × 10 cm in size as shown in Figure 1. An assessment of recurrent fibrosarcoma was made. Packed cell volume (PCV) was 17%. Chest X-ray showed cannonball metastases in the lungs with minimal right pleural effusion as shown in Figure 2. Abdominopelvic ultrasound was normal. The patient was resuscitated with 4 pints of blood pre-operatively (he had the rare O negative blood group); the anaesthetic assessment was ASA V. The mass was excised with the tumour weighing 5.7 kg. Postoperatively, the skin defect measured about 45 × 40 cm as shown in Figure 3a. He had 4 pints of blood transfused intra-operatively and was prepared for postoperative radiotherapy and skin cover with STSG. Six weeks postoperatively, he was discharged against medical advice because he could not afford further treatments. The postoperative skin defect had contracted significantly but now had metastatic nodules along the spine as shown in Figure 3b. Histology of the excised specimen confirmed fibrosarcoma.

## Discussion

Soft tissue sarcoma has no identifiable aetiology in most cases; while most of the tumours arise de novo, a variety of predisposing or associated factors have been identified. There are approximately 70 different histologic types of STS. Most sarcomas are classified according to the normal cell type they mimic, based on the system proposed by Enzinger and Weiss [2].

Malignant fibrous histiocytoma (MFH) (18%), liposarcoma (20%) and leiomyosarcoma (19%) constitute the majority of adult STS in western countries [3]. This is different from a report from Africa where fibrosarcoma (36.4%) appears to be the most common of the malignant soft tissue tumours [4]. They commonly present as an asymptomatic mass. Pain if present is usually mild and occurs later in the course of the disease. Thus, a patient might delay seeking medical attention and a definitive diagnosis might also be delayed [5]. This may be responsible for our patient presenting almost 14 years after the onset of the mass on his back. Even though the histological grade of the tumour excised was not reported by our pathologist, it is most likely to have been Grade 1 or Grade 2. The prognostic significance of grade and size of a STS varies over time. Grade is said to be the prime determinant of distant recurrence and death for the first 2 years. For later metastases, however, size is more important than grade [6]. The size of our patient's tumours (55/40/10 cm) coupled with the pulmonary metastases give him a very poor prognosis. During the first surgical excision in our hospital, the resection we did was R1 based on the R classification of the Union International Contra la Cancrum (UICC) [7]. However, at the second procedure, it was an R2 resection because tumour tissues were macroscopically obvious along the spine. In patients with sarcoma of the extremities and trunk, the 5-year survival rate is 57% and 15% for R0/1 and R2 resections, respectively [8]. The clinical condition of our patient was very poor before the last resection. The very low PCV of 17%, high pulse rate of 130/minute, low volume, sweating, pulmonary metastases and rare blood group (O negative) gave him a classification of ASA V. The anaesthetists were very reluctant to anaesthetize him. We had a pre-surgical conference which involved the surgical team, the anaesthetists, the Chief Nursing Officer (CNO) in charge of the operating theatre, and the parents of the patient. The clinical condition of the patient was presented and a decision was taken whether to go ahead with the surgical resection or not. This was crucial because any attempt to change the dressing of the tumour on the ward by the nurses is always accompanied by profuse bleeding. The possibility was that if the tumour was not resected and we had to wait for another 8 days to build him up, he could have bled so much as to go into anaemic heart failure and probably die, but also, if he was resected, there was the possibility of him not recovering from the anaesthetic.

At the end of the deliberation, it was agreed that the surgical excision should be undertaken. Post surgical excision, the recovery was uneventful; he was transfused with 4 pints of blood intra-operatively. In STS, patients considered to have a high risk of recurrence and death include those presenting with metastatic disease, localized sarcomas at sites other than the extremities, and sarcomas >5 cm of intermediate or high histologic grade [9]. All of these risk factors were present in our patient. The combination of surgical excision with postoperative radiation therapy has yielded improved local control rates of 78% to 91% [10]. Our patient would have benefited from postoperative radiotherapy. Even though we do not have the service in our centre, we normally send patients to another centre where the facility is available and which is at a distance of about 150 km. The parents of our patient could not afford to pay for any other service; they claimed to have sold all of their properties to get him to the stage we were at. The use of adjuvant chemotherapy was also discussed. There was no money to purchase the drugs even though their usefulness may be questionable in this type of patient. We were just left with the option of a twice weekly change of dressing. The issue of wound cover with split thickness skin graft could not be discussed. First, there was no money for the procedure and second, a recurrent tumour had started growing along the spine as shown in Figure 3b. The patient discharged himself against medical advice. Before discharge, the patient was freely ambulant unlike before, there was no more foul smelling discharge, no more contact bleeding and PCV was 28% with a fairly stable cardiovascular status. He will however probably succumb to metastases.

## Conclusion

In a developing country like ours where the poverty level is very high, there is no special provision available for patients with malignant lesions to access free treatment or to have their treatment highly subsidized by the government. If such provisions were available, many patients of low social class could have their clinical conditions well managed, thereby improving their overall survival rate.

## Consent

Written informed consent was obtained from the patient for publication of this case report and any accompanying images. A copy of the written consent is available for review by the Editor-in-Chief of this journal.

## Competing interests

The authors declare that they have no competing interests.

## Authors' contributions

JOB summarized the patient data and carried out an initial literature search on fibrosarcoma. IAA prepared the initial draft of the manuscript while KOO prepared the final draft. All authors participated in the patient's management, including the surgery. All authors read and approved the final manuscript.
